# Path Planning Strategies to Optimize Accuracy, Quality, Build Time and Material Use in Additive Manufacturing: A Review

**DOI:** 10.3390/mi11070633

**Published:** 2020-06-28

**Authors:** Jingchao Jiang, Yongsheng Ma

**Affiliations:** 1 Department of Mechanical Engineering, University of Auckland, Auckland 1142, New Zealand; jjia547@aucklanduni.ac.nz; 2 Digital Manufacturing and Design Center, Singapore University of Technology and Design, Singapore 486842, Singapore; 3 Department of Mechanical Engineering, University of Alberta, Edmonton, AB T6G 2V4, Canada

**Keywords:** additive manufacturing, path planning, review

## Abstract

Additive manufacturing (AM) is the process of joining materials layer by layer to fabricate products based on 3D models. Due to the layer-by-layer nature of AM, parts with complex geometries, integrated assemblies, customized geometry or multifunctional designs can now be manufactured more easily than traditional subtractive manufacturing. Path planning in AM is an important step in the process of manufacturing products. The final fabricated qualities, properties, etc., will be different when using different path strategies, even using the same AM machine and process parameters. Currently, increasing research studies have been published on path planning strategies with different aims. Due to the rapid development of path planning in AM and various newly proposed strategies, there is a lack of comprehensive reviews on this topic. Therefore, this paper gives a comprehensive understanding of the current status and challenges of AM path planning. This paper reviews and discusses path planning strategies in three categories: improving printed qualities, saving materials/time and achieving objective printed properties. The main findings of this review include: new path planning strategies can be developed by combining some of the strategies in literature with better performance; a path planning platform can be developed to help select the most suitable path planning strategy with required properties; research on path planning considering energy consumption can be carried out in the future; a benchmark model for testing the performance of path planning strategies can be designed; the trade-off among different fabricated properties can be considered as a factor in future path planning design processes; and lastly, machine learning can be a powerful tool to further improve path planning strategies in the future.

## 1. Introduction

Additive manufacturing (AM) technologies (also known as rapid prototyping, 3D printing, solid freeform fabrication, etc.) have been developed for more than 30 years [1,2,3,4,5,6]. As AM has matured, it has been applied in many fields, including aerospace, medical, construction and aesthetic products [7,8,9]. The manufacturing process of AM is different from conventional subtractive manufacturing, which uses a subtractive manner (e.g., tooling and cutting), while AM uses an additive process to fabricate parts from the bottom to the top in a point-by-point and then layer-by-layer strategy [10,11,12,13,14]. Currently, AM technology is mainly divided into seven categories: material extrusion, material jetting, powder bed fusion, binder jetting, vat photopolymerization, directed energy deposition and sheet lamination [15,16,17]. Among these seven AM techniques, material extrusion, material jetting, powder bed fusion, binder jetting, vat photopolymerization and directed energy deposition need a deposition path moving along the 3D model to fabricate the final product. The strategy of designing the paths for manufacturing is called path planning in AM. Path planning is very critical in AM, as different path strategies can affect surface roughness, dimensional accuracy and the properties (e.g., strength) of the printed products. In addition, different paths mean different moving strategies of the corresponding print head (e.g., the nozzle in fused deposition modeling (FDM), the laser in direct energy deposition (DED)), resulting in different durations needed for completing the same part. Therefore, a better path planning strategy can lead to better fabricated properties, qualities or a lower fabrication time. Currently, there are already a lot of papers that have been published on this topic. As a rapidly evolving manufacturing technology, new additive manufacturing technologies are being developed and will most likely continue to be developed in the future. It is, therefore, perhaps timely that a review of the topic with regard to path planning is performed, so that newly developed additive manufacturing technologies can exploit the most apposite strategies with better performance. The aim of this article is to provide a contextual framework for the range of research that has been carried out in the area of path planning for additive manufacturing. The article then reviews the differing strategies of path planning, based on different objectives. Finally, it gives an outlook onto potential future research directions.

## 2. Path Planning Strategies

**Figure 1:**
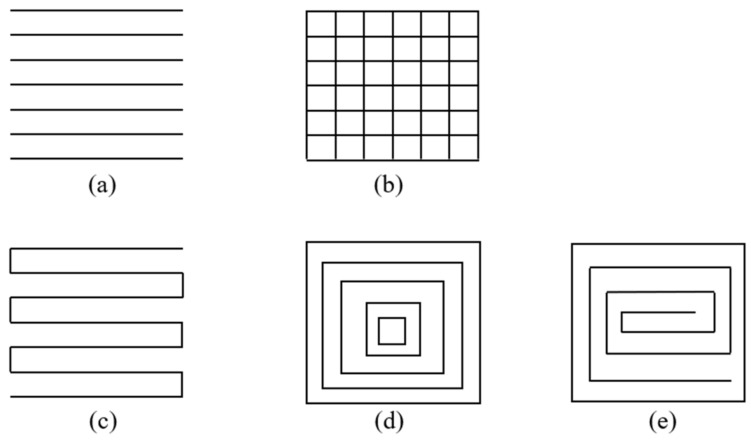
(**a**) Raster path; (**b**) grid path; (**c**) zigzag path; (**d**) contour offset path; (**e**) spiral path.

In this section, the current available path planning strategies are divided into three groups for illustration (i.e., improve printed qualities, save materials/time and achieve objective printed properties). “Improve printed qualities” means proposing path planning strategies in AM to improve printed surface quality (e.g., surface roughness), shape accuracy and infill distribution quality. “Save materials/time” means proposing path planning strategies in AM to save the total fabrication time or the material consumption. “Achieve objective printed properties” means proposing path planning strategies in AM to achieve better mechanical, topological or functional properties. Before going into detail, some commonly used path patterns are introduced first. Figure 1 shows the corresponding basic path patterns are currently being widely used. Most of the improved path planning strategies are based on these basic path patterns. These patterns are widely used in AM to fill the layers of sliced 3D models. These patterns can be selected easily in commonly used slicing software, such as Cura and Slic3r.

### 2.1. Improve Printed Qualities

**Figure 2:**
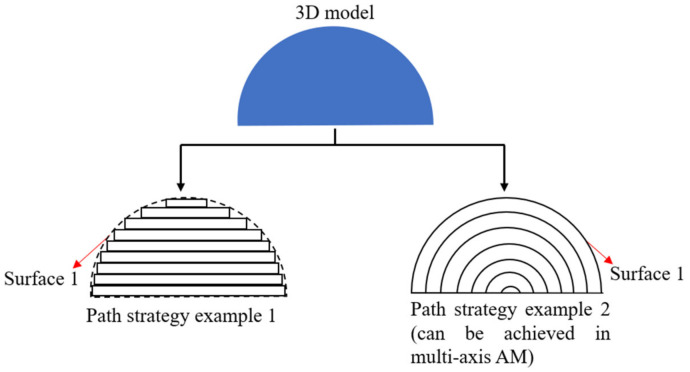
Illustration of path strategies influencing surface quality.

In this sub-section, path planning for improving printed qualities is illustrated (including printed surface quality, shape accuracy and infill distribution quality). Due to the point-by-point, line-by-line and layer-by-layer nature of AM, the qualities of printed parts generally deteriorate as there are gaps between the printed lines. This leads to different surface qualities, shape accuracies and infill distribution qualities in different path strategies. Figure 2 illustrates this phenomenon using two different path planning strategies. A 3D model can be manufactured in many different path strategies, Figure 2 only shows two examples. As can be seen, the surface qualities on Surface 1 in Figure 2 are probably different in these two different path planning strategies, as well as shape accuracies and infill distribution qualities.

**Figure 3:**
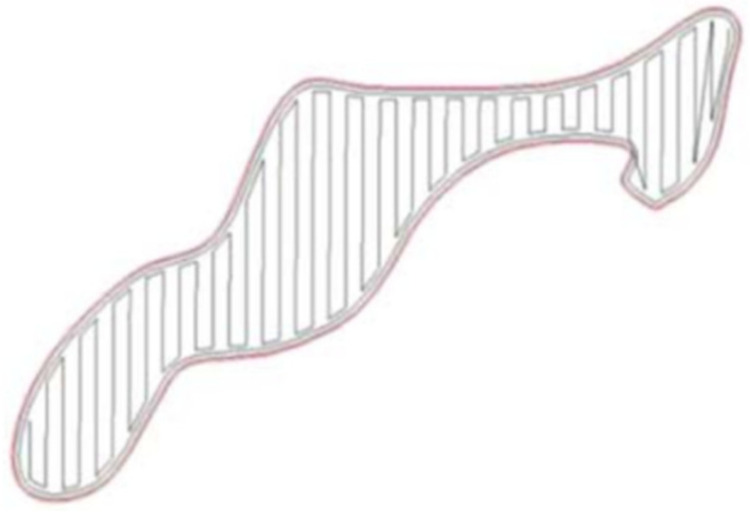
Example of a generated contour reproduced with permission from [18]. Elsevier, 2011.

Surface quality: Jin et al. [18,19] proposed to use closed non-uniform rational B-spline (NURBS) curves to represent the contours of layers to maintain the surface accuracy of the 3D part model. Then a mixed and adaptive path generation algorithm was developed to optimize the surface quality. This algorithm could generate contour paths for AM fabrication to reduce the surface errors of 3D models. Figure 3 shows an example of generated contour paths by using their method. A curved layer path planning method for traditional FDM processes was introduced by Jin et al. [20]. This method moved the path along a curved layer to improve the printed surface qualities. Similarly, Ezair et al. [21] also proposed a curved layer path planning strategy that could improve the surface quality, using volumetric covering print paths for material extrusion AM. Jensen et al. [22] proposed two path planning methods (path projection and parent–child approach) for five degrees of freedom (5DOF) and 6DOF material extrusion AM, respectively. Their strategy could remedy the staircase effect (shape deviations), thus achieving better surface quality.

**Figure 4:**
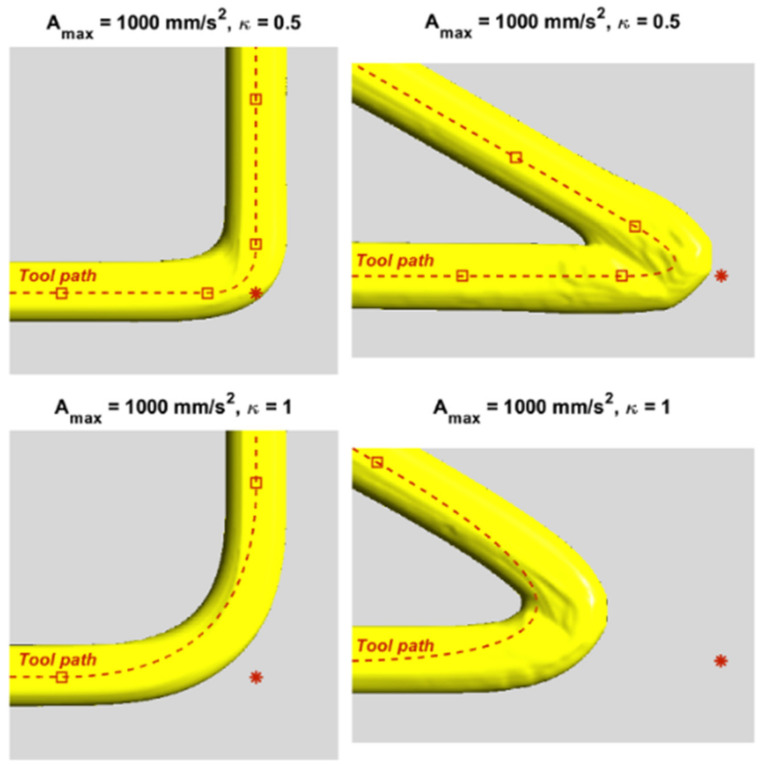
Successful fabrication of 90° and 30° corners reproduced with permission from [25]. Elsevier, 2019.

Shape accuracy: Routhu et al. [23] improved the zigzag and offset pattern based on the laser scanning speed to reduce the printed height variation in the laser powder-based metal deposition process, achieving better shape accuracy. A three-step path planning strategy was developed by Jin et al. [24] to achieve precision manufacturing in FDM. Communal et al. [25] proposed a path planning strategy considering the shape accuracy of the corners in each layer in material extrusion AM. Figure 4 shows their successful fabrication of corners with good quality. Liu et al. [26] developed a composite path planning method with a sharp corner correction strategy to improve the shape accuracy of parts fabricated in wire and arc AM. Giberti et al. [27] proposed a path planning algorithm based on the use of Bézier curves aimed at assuring the regulation of the velocity and a uniform distribution of the extruded material in binder jetting AM, thus improving fabricated shape accuracy. Ding et al. [28] introduced an automatic path planning method for wire and arc AM, which can achieve good shape accuracy of fabricated parts.

Infill distribution quality: A contemporary path planning strategy was proposed by Eiliat and Urbanic [29,30,31], to find optimal paths for achieving better infill quality without voids. Xiong et al. [32] developed a variable bead width path planning method to manufacture void-free parts in a wire and arc AM process. In their strategy, the path planning process considers the possibility of changing bead width when fabricating. In the traditional constant offset path method, voids are left in the middle of each layer, while by using their proposed adaptive offset path, the manufactured parts can achieve better infill distribution without voids. Kumar and Maji [33] also proposed a path planning method to optimize the path width and overlapping between two beads to achieve void-free parts in wire and arc AM. However, they did not consider the changeable bead widths in each path planning. Wang et al. [34] proposed a cylindrical path planning strategy to fabricate cylindrical parts (such as the blades of a propeller), without voids inside, using wire and arc AM. A sequential path planning strategy for wire and arc AM was proposed by Wang et al. [35], based on a water-pouring rule. Their proposed solution can transfer all the intersection areas of the path to the outer contour, ensuring that the inner area is uniform and compact. Michel et al. [36] introduced a modular path planning (MPP) strategy that incorporates the modularity of feature-based design into the conventional layer-by-layer method. Their strategy can ensure a uniform defect-free deposition in wire and arc AM processes. A medial axis transformation (MAT)-based path planning strategy was developed by Ding et al. [37,38,39], to allow the wire and arc AM to deposit material along multiple directions. Their MAT-based paths can guarantee the void-free deposition of layers. They further developed a new path planning strategy specifically for thin-walled parts without voids inside [40]. Ren et al. [41] proposed a path planning strategy of combining a contour-parallel pattern and adaptive zigzag path pattern to achieve void-free part fabrication in metal deposition AM. Han et al. [42] used a grouping and mapping algorithm to generate paths for fabricating parts without voids inside in FDM processes. Their path planning strategy was based on normal zigzag and contour paths with a better calculated distance between each path line, resulting in void-free infill. Jin et al. [43,44] developed an FDM path generation method that chooses a better inclination of paths to reduce the number of sharp corners in layers, so this method can adaptively generate paths for fabrication with better infill quality. 

### 2.2. Save Materials/Time

In this sub-section, path planning for saving materials or fabrication time is presented.

**Figure 5:**
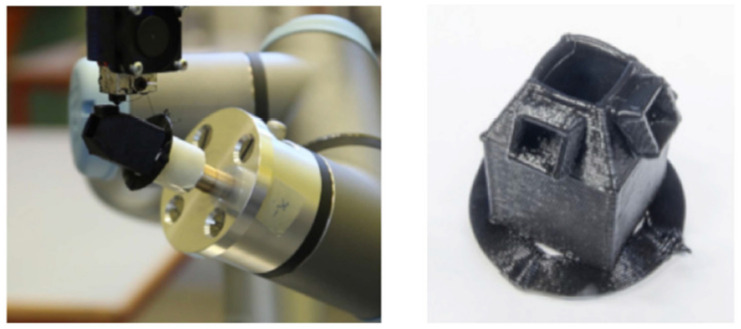
5DOF AM machine (**left**) and the product fabricated without any support (**right**) [22].

Save materials: Jensen et al. [22] proposed two path planning methods (path projection and parent–child approach) for five degrees of freedom (5DOF) and 6DOF material extrusion AM, respectively. Their strategy can achieve successful fabrication without support structures, thus saving support material consumption. Figure 5 shows the example 5DOF machine and the product fabricated using their path planning method. Zhao et al. [45] introduced a nonplanar path planning strategy that can reduce the usage of support material in robot-based material extrusion AM. Tarabanis [46] developed a path planning strategy, based on shelving and bridging features, that allows parts to be able to be printed “in the air” in FDM, thus reducing support structure usage. We also previously proposed path planning strategies with the aim of reducing support material consumption in FDM [47,48,49,50]. Nguyen et al. [51] proposed a heuristic path planning strategy for wire and arc AM that can achieve support-free fabrication, thus reducing material waste. The path planning method, based on medial axis transformation (MAT) and developed by Ding et al. [37,38], can also save material usage in wire and arc AM. Thompson and Yoon [52] developed a path planning algorithm to control the motion of an XY stage in aerosol printing (material jetting) for an arbitrary printing path and desired velocity while minimizing material waste. A path planning strategy for an eight-axis direct energy deposition system was proposed by Ding et al. [53] to fabricate complex revolved parts without support consumption. Zhang and Liou [54] developed an automated path planning strategy for five-axis laser aided AM that can reduce the usage of support structures.

**Figure 6:**
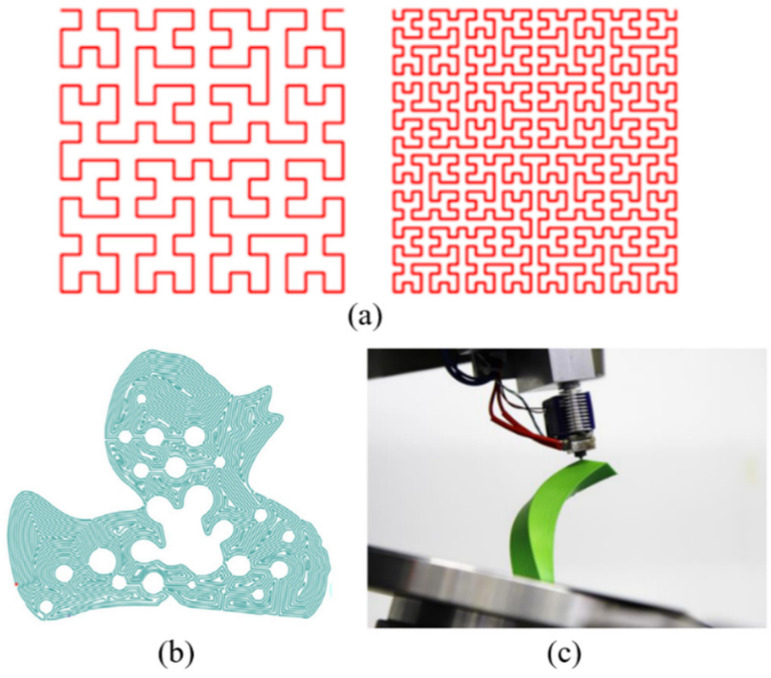
(**a**) Hilbert curves path pattern reproduced with permission from [62]. Elsevier, 2018; (**b**) an example of generated paths for porous structures reproduced with permission from [65]. Elsevier, 2019; (**c**) a successful fabrication by using the path planning strategy reproduced with permission from [67]. Elsevier, 2018.

Save time: Bui et al. [55] proposed a path planning strategy for multi-head material extrusion AM, where multiple printheads can work together without collision, thus reducing fabrication time. Their strategy is based on multiple heads printing the same material. Choi and Zhu [56] proposed a dynamic priority-based path planning strategy for multi-material extrusion AM with multiple nozzles. Their strategy can generate optimized paths for different nozzles (with different materials) and avoid collisions between nozzles, thus saving total fabrication time. A combined heuristic path planning method was proposed by Volpato et al. [57] to reduce the total moving length of the extruder nozzle in material extrusion AM, thus saving fabrication time. Ganganath et al. [58] introduced a path planning method using triangular and trapezoidal velocity profiles for material extrusion AM. Their method can generate optimal paths to minimize the transition time between print segments. Fleming et al. [59] proposed a continuous path planning strategy that can reduce the distance traveled between subsequent space-filling curves and layers, which reduces unnecessary nozzle movement by around 20%. The closed non-uniform rational B-spline (NURBS) path planning method from [18,19] can also minimize the build time through their developed analysis mathematical models. Fok et al. [60] developed a path planning strategy based on the Christofides algorithm that can significantly reduce the length of motion paths in FDM, compared to a nearest neighbor-based strategy. Jin et al. [61] developed a non-retraction path planning strategy that can avoid retraction during the printing process in FDM, and hence the time spent moving along these retracting paths can be saved. Papacharalampopoulos et al. [62] proposed a path planning strategy that ensures a single continuous motion of the printhead to finish a printing in material extrusion AM. They used the Hilbert curves as the path pattern, as shown in Figure 6a. Luo and Tseng [63] proposed a path planning strategy for multi-part production in FDM. They tried to reduce the length of paths traveled between parts to reduce the fabrication time. Jiang proposed a multi-layer by multi-layer path strategy to save the fabrication time [64]. A porous path planning strategy was introduced by Zhai and Chen [65] for the successful fabrication of porous structures in material extrusion AM. Figure 6b shows a result of their generated paths also shown in this figure. Their strategy found the optimal workable paths that could save time for printing porous structures. Dreifus et al. [66] proposed a path planning strategy, based on the Chinese postman problem, specifically for fabricating lattice structures that can minimize total manufacturing time in material extrusion AM. Coupek et al. [67] proposed a path planning method for seven-axis material extrusion AM which can avoid the usage of supports, thus saving materials and fabrication time as well. Figure 6c shows a successful fabrication using their strategy in their seven-axis FDM machine. An efficient path planning strategy was developed by McQueen et al. [68] for material extrusion AM with two robotic arms. The allowed two robotic arms working together could save fabrication time. Shembekar et al. [69] proposed a collision-free path planning strategy for a 6DOF material extrusion AM system, which could save both material usage and build time. A group-based path planning strategy for a multi-robotic material extrusion AM system was developed by Cai and Choi [70]. Their strategy could ensure collision-free printing between printheads, thus saving total fabrication time when all the robotic heads work together. For wire and arc AM, Fügenschuh et al. [71] proposed a path planning method on how to partition a given traverse into continuous segments that are printed without intersection and deadheading between two segments, so that deadheading is minimized to complete the fabrication as fast as possible.

### 2.3. Achieve Objective Printed Properties

In this sub-section, path planning for achieving objective printed properties is presented (including mechanical, topological, functional, etc.).

Li et al. [72] developed an ingenious path planning strategy that can print continuous carbon fiber-reinforced composites with complex shapes and high mechanical performances in material extrusion AM. Asif [73] also proposed another strategy with the same aim in material extrusion AM. Kraljić and Kamnik [74] developed a path planning strategy that can enhance inter-track bonding and consequently better strength of printed parts in 6DOF material extrusion AM. Liu et al. [75] proposed a path planning strategy for achieving topologically optimized lightweight part fabrication in FDM. They also proposed a path planning strategy along the principle stress direction of parts to enhance the structural performance of FDM-printed parts [76]. Wavy path planning was developed by Jin et al. [77] to improve the structural strength of printed parts in FDM. Lin et al. [78] proposed a maze-like path planning strategy that could fabricate isotropic components in FDM. Jin et al. [79] developed a path planning strategy for the successful fabrication of thin-walled parts with good qualities in FDM. Ma et al. [80] also proposed an adaptive path planning method with varying thickness to successfully fabricate thin-walled parts, but using wire and arc AM. Eliseeva et al. [81] developed a path planning strategy for the successful fabrication of functionally graded compositions in multi-material direct energy deposition systems. Deuser et al. [82] also proposed a path planning method that can successfully print functionally graded compositions, but in material extrusion AM systems with three printheads. Ozbolat and Khoda [83] proposed a simulation-based path planning strategy to determine the sequence of material deposition in AM, achieving the successful fabrication of hollow porous structures with functionally graded materials. Zhu and Yu [84] developed a path planning strategy, based on a dexel-based spatio-temporal modeling approach that can guarantee the collision-free movement of printheads, achieving multi-material printing simultaneously in FDM with multiple printheads. We previously proposed a support interface path planning strategy for easy part removal after fabrication in direct energy deposition processes [85].

## 3. Discussion

Table 1 lists the available path planning strategies in literature based on different objectives. In the future, the corresponding path planning strategy can be selected according to the requirements of the products. For example, when a product with great mechanical strength is required, then the five available choices can be the candidates. Further, the 6DOF or normal material extrusion AM machines can be selected upon the availability of AM machines. Another example is that if the surface quality is a priority, then the six strategies listed in “improve surface quality” in Table 1 can be considered. However, it is hard to say which strategy is better than another among these six strategies, as the different strategies used different parts, standards or criteria. 

Another thing that needs to be known is that other properties or qualities may deteriorate when adopting an improved path planning strategy for a specific aim. For instance, when trying to improve the strength of printed parts by using a different path pattern, the dimensional variation and/or building time of parts may probably also be changed. The trade-off among these should be considered.

**Figure 7:**
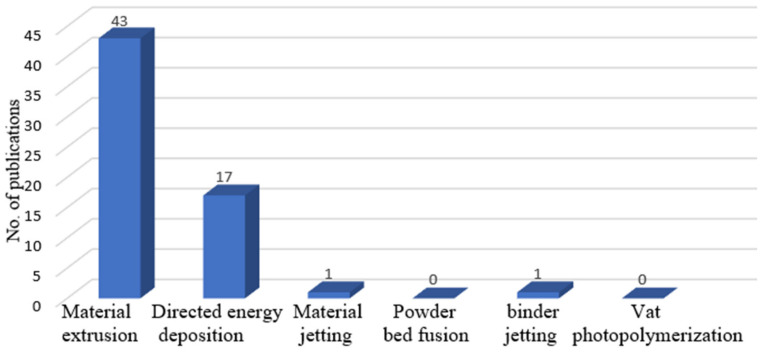
Number of publications on path planning in the seven AM categories.

**Figure 8:**
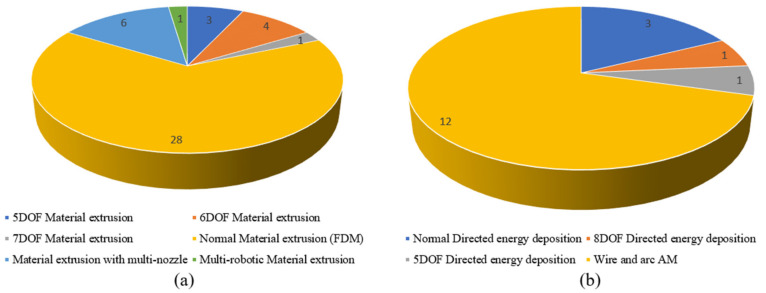
Distribution of published papers in material extrusion (**a**) and directed energy deposition (**b**).

Looking at the number of publications on path planning in the seven AM categories (as shown in Figure 7), material extrusion AM and directed energy deposition attracted most of the attention from researchers. Only one publication is seen on optimizing paths for material jetting AM and one for binder jetting AM. There are no published papers on path planning in the other two categories. When looking deeper into material extrusion and directed energy deposition, as shown in Figure 8, most publications (28 papers) focused on FDM in material extrusion while most publications (12 papers) focused on wire and arc AM in directed energy deposition. One of the reasons for this is that material extrusion (especially FDM) and directed energy deposition are the two most commonly used and have been applied in many fields in our daily lives. In addition, FDM is the simplest and most economical AM technique which mainly uses polymers as raw material with low costs. The principles and findings of path planning research on FDM, to some extent, can be extended to be used in other AM techniques, such as directed energy deposition, which always costs a lot. As directed energy deposition has its specific advantages, especially its ability to manufacture metal lightweight parts, it has been increasingly applied in aeronautic and astronomic fields. Therefore, increasing attention is also paid to directed energy deposition. As can be seen from Figure 8, there are also some publications on multi-DOF AM systems. This is mainly due to the fact that traditional three-DOF AM has some limitations (such as the staircase effect on surface finishes, anisotropy of parts, limited mechanical strength and the requirement of support structures). Multi-DOF AM systems are currently being investigated to overcome these disadvantages. 

**Figure 9:**
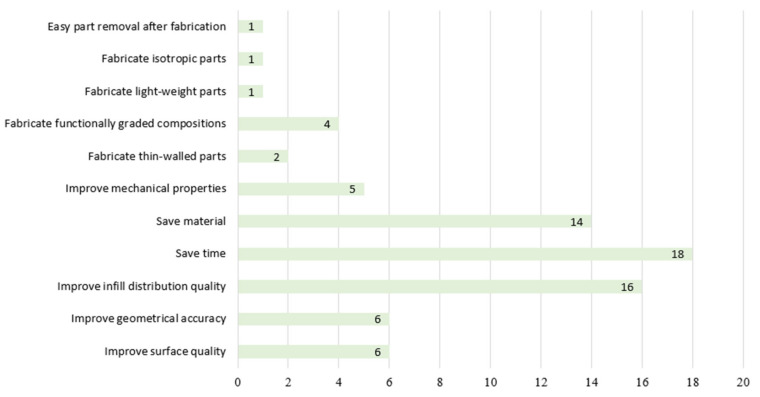
Number of publications with different objectives.

Looking into the objectives of path planning (Figure 9), researchers were concerned mostly about saving time (18 papers), saving material (14 papers) and improving infill distribution quality (16 papers). This indicates that path planning has a great contribution to the corresponding time spent on fabrication, material consumption and infill quality. Path planning is important in ensuring AM parts meet the required qualities. There are also some papers focusing on achieving specific properties (e.g., functionally graded compositions). This means path planning can help to broaden the application of AM in more fields in the future. When considering improving the performance of printed parts in different applications using AM, developing novel path planning strategies may be helpful.

## 4. Future Perspectives

**Figure 10:**
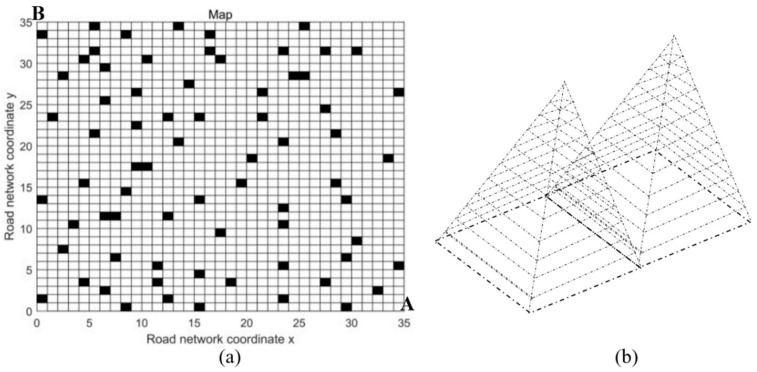
(**a**) Problem map for selecting the best path reproduced with permission from [102]. IEEE, 2019; (**b**) example of a 3D model divided into points for path selection using machine learning.

(1) As discussed above, path planning strategies on saving materials or fabrication time have been explored a lot. However, few studies can be seen considering the energy consumption in different path planning strategies. As the world is becoming more sustainable and the focus on sustainable manufacturing increases significantly, AM can become more sustainable and environmentally friendly in the future through path planning. Research on proposing novel path planning strategies that can save more energy can be a meaningful research topic in the future.

(2) Develop new AM systems based on the knowledge of the abovementioned path planning strategies. The current available path planning strategies have been summarized in Table 1, which can help engineers to choose how to combine their strategies for new AM system development. For example, if developing an AM system consisting of 10 robots working together to print a house, the path planning process for this new system can borrow ideas from the path planning strategy for AM with two robotic arms [68]. Another example is for developing a new AM system with three printheads, then combining the proposed methods of “multi-head strategy” [55] and “wavy strategy” (improved mechanical properties of printed parts) [77] can obtain a better AM system with better performance of fabricated parts.

(3) A comprehensive path planning strategy that can deal with all the aims (quality, function, time/material minimization, etc.) or some of the aims might be able to be developed in the future, based on the current available knowledge listed in Table 1. For example, considering both the surface roughness and mechanical strength as the objective, then it is possible to combine two path planning strategies (one from improving surface roughness and one from improving mechanical properties) together and revise them into a new path planning strategy to achieve the corresponding aims. In addition, while current path planning strategies are generally only suitable for a specific AM technique, a path planning strategy that can be used in many AM techniques rather than just one (such as only FDM) might be able to be developed in the future.

(4) A path planning platform can be developed in the future. This platform can automatically help to choose the best path planning strategy based on the required input properties. This platform should know all the advantages and disadvantages of each path planning strategy and which path planning strategy for which AM technique. Then, when inputting the objectives (e.g., saving time), the corresponding available strategies and recommended best strategy will pop up, with advantages and disadvantages.

(5) As discussed previously, other properties or qualities may deteriorate when adopting an improved path planning strategy for a specific aim. For instance, when trying to improve the strength of printed parts by using a different path pattern, the dimensional variation and/or building time of parts may also probably be changed. The trade-off among these can be investigated in the future. The trade-off depends on the specific requirements from the customers. For example, if the customer would like to have his/her product as soon as possible with a mechanical strength requirement, then the fabrication time is a priority, while the mechanical strength only needs to be at the qualified level. Based on this, a better path planning strategy can be selected or proposed.

(6) Currently, it is not possible to distinguish which path planning strategy is better than another, as these strategies use different parts/models in the research. A benchmark model with all the necessary features (sharp corner features, thin-walled features, etc.) can be developed in the future for comparing different path planning strategies. Once this benchmark model is available and all the upcoming research studies can be carried out based on this benchmark model, then it will be possible to know which path planning strategy is better than another in terms of some objectives (e.g., surface roughness). This will help to provide useful information for the future choice of adopting which path planning strategy. For example, when fabricating thin-walled parts, choose the path planning strategy that is best for thin-walled features.

(7) As the development of AM systems, new AM techniques are emerging rapidly. The hybrid AM system including additive and subtractive processes with multi-axis machines is one of these new developed AM techniques. Research on path planning for these new advanced AM systems is necessary in the future for its further development. 

(8) Machine learning, integrated path planning strategies, can be developed in the future. Machine learning is one of today’s most rapidly growing technical fields. It is a subset of artificial intelligence, mainly focusing on using algorithms and statistical models to make decisions without specific programming. Generally, machine learning can be used in medical diagnosis, image processing, prediction, classification, etc. Recently, research on using machine learning in AM has also been published for AM process optimization [86,87,88,89,90,91], dimensional accuracy analysis [92,93,94,95], manufacturing defect detection [96,97,98] and material property prediction [99,100,101]. However, machine learning has not been applied to improving path planning strategies yet. In fact, machine learning is very powerful in planning strategies. Liu et al. [102] used machine learning to select the best path for driving cars with the shortest path length. Figure 10a shows the problem map in their study, the black grids are the places where there are obstacles. The start point is A, while the destination is point B. In their study, machine learning solved this problem efficiently. Similarly, in the path planning problems of AM, machine learning can also be used to obtain the best paths and print sequences. All the print paths in the AM fabrication process can be divided into points (Figure 10b) and the print order of the points can be decided through machine learning. 

## 5. Conclusions

Path planning is an important step of AM fabrication that can influence the final printed properties, qualities, etc. Most of the research done in AM focuses on improving AM processes, the development of new AM techniques, and new applications of AM, based on the commonly used path strategies. However, there are still many researchers try to improve AM with different objectives through developing new path planning strategies. In this paper, the focus is given to these publications on path planning. A comprehensive review on path planning strategies is provided according to the aims of improving printed qualities, saving materials/time and achieving objective printed properties. A summarized table is provided for selecting suitable path planning strategies in future AM fabrication with specific aims. The main finding of this review is that there is still plenty of research on path planning that can be carried out in the future. New path planning strategies can be developed by combining some of these strategies (Table 1) with better performance. A path planning platform can be developed to help select the most suitable path planning strategy with required properties. Research on path planning, considering energy consumption, can be carried out in the future. A benchmark model for testing the performance of path planning strategies can be designed. The trade-off among different fabricated properties can be considered as a factor in future path planning design processes. Lastly, machine learning can be a powerful way to further improve path planning strategies.

## Figures and Tables

**Table 1:** Path planning strategies in literature.

Objective	Path Planning Strategy	Suitable AM Technique
Improve surface quality	NURBS-based strategy [18,19]	Material extrusion
	Curved layer strategy [20]	FDM (material extrusion)
	Curved layer strategy [21]	Material extrusion
	Path projection strategy [22]	5DOF material extrusion
	Parent–child approach strategy [22]	6DOF material extrusion
Improve shape accuracy	Improved zigzag/offset strategy [23]	Laser powder-based metal deposition process (directed energy deposition)
	Combination of zigzag and contour pattern strategy [28]	Wire and arc AM (directed energy deposition)
	Three-step strategy [24]	FDM (material extrusion)
Improve shape accuracy for corners	Corner strategy [25]	Material extrusion
	Composite strategy [26]	Wire and arc AM (directed energy deposition)
Improve shape accuracy under velocity constraints	Bézier curve strategy [27]	Binder jetting
Improve infill distribution quality	Contemporary strategy [29,30,31]	Material extrusion
	Variable width strategy [32], optimized width and overlapping strategy [33], water-pouring strategy [35], modular strategy [36], MAT strategy [37,38,39]	Wire and arc AM (directed energy deposition)
	Cylindrical strategy specifically for cylindrical parts [34]	
	MAT strategy specifically for thin-walled structures [40]	
	Grouping and mapping strategy [42], sharp corner strategy [43,44]	FDM (material extrusion)
	Adaptive contour/zigzag strategy [41]	Metal-directed energy deposition
Save time	Dynamic priority-based strategy [56]	Multi-material extrusion with multiple nozzles (material extrusion)
	Multi-head strategy [55]	Multi-head material extrusion
	Two-robot strategy [68]	Material extrusion with two robotic arms
	Combined heuristic strategy [57], salesman strategy [58], continuous strategy [59], NURBS-based strategy [18,19], Christofides strategy [60], Hilbert curve strategy [62], non-retraction strategy [61]	Material extrusion
	Porous strategy specifically for porous structures [65]	Material extrusion
	Lattice strategy specifically for lattice structures [66]	Material extrusion
	Multi-part strategy specifically for multi-part production [63]	FDM (material extrusion)
	Partition strategy [71]	Wire and arc AM (directed energy deposition)
	Collision-free strategy [69]	6DOF material extrusion
	Coupek strategy [67]	7DOF material extrusion
	Group-based strategy [70]	Multi-robot material extrusion
Save material	Heuristic strategy [51], MAT strategy [37,38]	Wire and arc AM (directed energy deposition)
	Aerosol strategy [52]	Material jetting
	Nonplanar strategy [45]	5DOF material extrusion
	Support optimization strategy [47,48,49], shelving- and bridging-based strategy [46]	FDM (material extrusion)
	Path projection strategy [22]	5DOF material extrusion
	Five-axis adaptive slicing strategy [54]	5DOF directed energy deposition
	Parent–child approach strategy [22], Collision-free strategy [69]	6DOF material extrusion
	Revolved strategy [53]	8DOF directed energy deposition
Improve mechanical properties	Ingenious strategy for fiber-reinforced fabrication [72], Asif strategy for fiberreinforced fabrication [73], stress direction strategy [76]	Material extrusion
	Kraljić strategy [74]	6DOF material extrusion
	Wavy strategy [77]	FDM (material extrusion)
Fabricate thin-walled parts	Varying thickness strategy [80]	Wire and arc AM (directed energy deposition)
	Wavy strategy [79]	FDM (material extrusion)
Fabricate functionally graded compositions	Functional strategy [81]	Directed energy deposition
	Simulation-based strategy [83]	AM
	Spatio-temporal strategy [84]	FDM with multiple nozzles (material extrusion)
	Deuser strategy [82]	Material extrusion with three nozzles
Fabricate lightweight parts	Topology strategy [75]	FDM (material extrusion)
Fabricate isotropic parts	Maze-like strategy [78]	FDM (material extrusion)
Easy part removal after fabrication	Support interface strategy [85]	Directed energy deposition
